# Comparison of physical characteristics among english professional and semi-professional soccer players across different leagues

**DOI:** 10.1371/journal.pone.0324436

**Published:** 2025-05-29

**Authors:** Nicholas Joel Ripley, Jack Fahey, Paul Jones, Jake Batsford, Paul Sindall, Christopher Bramah, Paul Comfort

**Affiliations:** 1 Directorate of Psychology and Sport, University of Salford, Salford, Greater Manchester, United Kingdom; 2 Manchester Institute of Health and Performance, Nuffield Health, Epsom, Surry, United Kingdom; 3 Strength and Power Research Group, School of Medical and Health Sciences, Edith Cowan University, Joondalup, Australia; Afyon Kocatepe University: Afyon Kocatepe Universitesi, TÜRKIYE

## Abstract

The purpose of the present study was to investigate if differences exist in neuromuscular qualities between different leagues in English male soccer. Twenty soccer players (age: 23.3 ± 5.2 years, stature: 180.3 ± 7.0 cm, mass: 82.5 ± 7.7 kg) from English football league two (EFL2). 34 soccer players (age: 25.8 ± 4.3 years, stature: 180.9 ± 5.6 cm, mass: 81.4 ± 8.6 kg) from National league (NL) and 23 soccer players (age: 27.5 ± 4.3 years, stature: 182.1 ± 5.5 cm, mass: 84.1 ± 8.0 kg) from National league North (NLN). Each player completed three to five repetitions of the countermovement jump (CMJ), countermovement rebound jump (CMJRJ) and isometric mid-thigh pull (IMTP). Trivial to small differences were observed in CMJ jump height, CMJ momentum, relative average braking and propulsion force, time to take-off and modified reactive strength index (RSI) (*p *> 0.265, *d* = 0.05–0.55). However, moderate to large (*p* ≤ 0.009, *d* = 0.94–1.25) differences were observed in countermovement depth. Trivial to moderate differences were observed in absolute and relative peak net force and force at 100 and 250 ms (*p *≥ 0.092, *d* = 0.13–0.63). Trivial to moderate differences were observed in CMJRJ rebound jump height and rebound jump momentum (*p *≥ 0.440, *d* = 0.17–0.41). NLN players had longer rebound contact time and lower rebound RSI, rebound average relative braking and propulsion forces to a large magnitude (*p* ≤ 0.001, *d* = 1.18–1.85), with small differences between ELF2 and NL (*p* > 0.536, *d* = 0.11–0.39). Lower reactive qualities of NLN players could explain observed leagues, considering their relationship with match scoring situations. All players would be considered weak (<30 N/kg) and practitioners should prioritise lower limb strength across all leagues.

## Introduction

Sport-specific movements such as sprint acceleration, maximal velocity running, deceleration and change of direction are key aspects of athletic performance within soccer [1–3]. The quality and magnitude of these movements are influenced by a combination of physical, physiological [4] and technical qualities, alongside tactical knowledge and in-game variability. Exposure to complex, sport-specific movements has increased over time in elite player groups (4), and whilst a greater number of intensive sprints, decelerations and change of direction movements are frequently performed at higher levels of competition [5–7], there is evidence that the gap between mid- to high-ranking teams is narrowing (5). The trend for more frequent exposure to situations involving intense sport-specific movements places an emphasis on the requirement for enhancement of core physical qualities, to enable players to successfully cope with repeated demands of match-play from both an injury risk reduction and performance perspective. The need for well-developed physical qualities is likely to be more important when competing at higher tiers (i.e., leagues), as identified in Spanish soccer players [8], and therefore, quantification of observable differences is of considerable practical value. For example, acceleration is determined by relative force production (i.e., the force produced in relation to the mass of the object being accelerated) with the resulting velocity determined by the duration of acceleration, with maximal velocity actions considered crucial for scoring situations [1].

The results from several studies show that neuromuscular qualities such as ballistic, maximal and reactive strength are core physical attributes due to their relationship to performance during sprint acceleration, deceleration and change of direction tasks [9–14]. In particular, countermovement jump (CMJ) variables of peak propulsive force, peak braking force and peak propulsive power have been shown to differentiate between soccer players with high and low deceleration capabilities [13]. Furthermore, reactive strength index and propulsive mean force during a drop jump demonstrate strong associations with deceleration ability [12]. Isometric mid-thigh pull (IMTP) variables have also been shown to relate to change of direction speed and sprint acceleration performance [9]. Consequently, the testing and monitoring of neuromuscular qualities using force plates has become part of regular practice in soccer settings [15–17].

Considering the known association between force generating qualities and performance during sport-specific movements [9–14], it is anticipated that differences in neuromuscular and physical qualities will exist between players competing at different competitive tiers of the sport. This notion is supported by investigations focused on sports with similar movement demands and characteristics to those of soccer, such as rugby union and league, Gaelic football and Australian rules football, where elite groups have demonstrated greater neuromuscular strength qualities than lower-tier counterparts [18–21]. In soccer specifically, the literature is less extensive with limited information on the differences in the physical qualities amongst teams competing at different levels within the league system [8]. Therefore, the purpose of this study was to investigate whether differences exist in neuromuscular qualities between soccer players competing at different levels in the lower tiers of English soccer (i.e., English football league 2 (EFL2), National league (NL) and National league North (NLN)). Specific objectives were to determine if differences exist within CMJ, countermovement rebound jump (CMJRJ) and IMTP force-time characteristics. It was hypothesized that a) soccer players competing at higher levels would exhibit greater performances in the CMJ, CMRJ and IMTP than lower-level counterparts, and that b) values would be consistent with the increases in IMTP peak force observed within Spanish soccer players at higher levels of competition [8].

## Materials and methods

An observational research design was used to assess ballistic, plyometric and isometric strength of the lower limb within professional and semi-professional soccer players. Each player completed a minimum of three but up to five repetitions of the CMJ, CMJRJ and IMTP. All testing was completed within the first week of pre-season in June 2024, with no intense training in the 72-hours prior to testing. Written and informed consent was sought from all players prior to testing along with a participant readiness health questionnaire. Ethics approval was granted by the School of Health and Society at the University of Salford (ID 2216), conforming to the Declaration of Helsinki (2013).

A priori sample size estimation was performed for the present study, using the identified effect size from Soriano et al. [8] who compared IMTP force values between national and regional Spanish soccer players for IMTP force production (η_p_^2^ = 0.109, *f* effect size = 0.35). Using alpha error probability of 0.05, statistical power 80% and comparing between three groups, a minimum sample size of 84 participants was identified

### Participants

Participants were recruited from the lower tiers of the English soccer league system (recruitment commenced on the 4^th^ March 2024, testing commenced on the 27^th^ June 2024 and was completed by 4^th^ July 2024), incorporating athletes from one EFL2 squad, two NL squads and one non-league squad in the NLN. Twenty male soccer players (age: 23.3 ± 5.2 years, stature: 180.3 ± 7.0 cm, mass: 82.5 ± 7.7 kg) were from EFL2 (fourth tier in English soccer, finished 85^th^ across English soccer 2023–24 season), 34 male soccer players (age: 25.8 ± 4.3 years, stature: 180.9 ± 5.6 cm, mass: 81.4 ± 8.6 kg) from NL (fifth tier in English soccer, finished 103^rd^ and 110^th^ across English soccer 2023–24 season) and 23 male soccer players (age: 27.5 ± 4.3 years, stature: 182.1 ± 5.5 cm, mass: 84.1 ± 8.0 kg) from NLN (seventh tier in English soccer, finished ~143^rd^ across English soccer 2023–2024 season).

### Procedures

All testing was carried out at one of four sites in the North West of England using club training facilities. On arrival, participants completed a standardized RAMP warm up, which included low intensity cardiovascular exercise (e.g., bike or jog), dynamic stretches and football-specific movements, including (but not limited to) squats, lunges, hops, and submaximal jumps. In all cases, warm up sessions were devised and administered by trained personnel from within the club’s strength and conditioning department. To mitigate order effects, tests were completed in a randomised sequence. Dual sensor portable force plates sampling at 1000 Hz (Hawkin Dynamics Inc., Maine, USA) were used for all testing, with both hardware and proprietary software found to be both valid and reliable [22,23]. Foam surrounds were placed around the force plates for participant safety during all jump testing, with force plates situated on solid even surface.

The IMTP was conducted in accordance with accepted protocols from Comfort et al. [24], with the posture replicating the start of the second pull phase of the clean. Weightlifting straps were used to eliminate grip strength as a limiting factor. Participants were required to remain as still as possible for at least 1-s to allow calculation of system mass. Strong verbal encouragement included “*push*” with consistent instruction provided for all participants (i.e., “*push as hard and as fast as possible for 3 to 5 seconds*.”). An additional trial was included if a difference of>250N was observed between trials; in this case, a 1-minute rest period was provided.

The CMJ, and CMJRJ were both completed with arms akimbo. Participants were required to stand on the centre of each force plate for a 1-s weighing period and were instructed to “*jump as high and as fast as possible*”, contextualized to the phase of the jump, for example, “*jump as fast*” refers to performing the countermovement, propulsive and rebound phases as quickly as possible. Trials involving displacement of the hands from their position on the hips were excluded from data analysis with a new trial performed after a 1-minute rest. CMJRJ was performed with the same instruction as the CMJ but with the added instruction to rebound “*as fast as possible*” upon landing from the initial CMJ portion of the task.

### Data analysis

Vertical ground reaction force was low-pass filtered at 50 Hz in accordance with recommendations [25], while take-off was determined when the vertical force dropped below 25 N during the propulsive phase. All metrics were calculated automatically by the force plate proprietary software [22]. The IMTP force-time data were analysed using an onset threshold of an increase in force >3 standard deviations of the force during the 1-s period of quiet standing, with the highest force achieved identified as peak force. System mass determined from the CMJ was subtracted from this value to ensure that only net peak force was reported. Relative metrics for IMTP were calculating using the body mass observed from the CMJ (i.e., IMTP relative peak force = net peak force divided by body mass).

### Statistical analyses

Normality of all data were confirmed via Shapiro-Wilk’s test, a two-way mixed effect, absolute agreement ICC (model 3,1) was used to assess relative reliability [26]. ICC values were interpreted based on the lower bound 95% confidence interval (ICC_-95_) as: poor <0.50, moderate between 0.50 and 0.74, good between 0.75 and 0.89, and excellent >0.90 using accepted thresholds for determination [26]. Absolute reliability was interpreted from the upper 95% confidence interval for the CV (CV_+95_) interpreted as: ≥ 15%, 10–15%, 5–10% and ≤5% (poor, moderate, good and excellent absolute reliability, respectively).

Discrete one-way analyses of variance (ANOVA) were performed to examine between-group differences (EFL2 vs. NL vs. NLN) for each of the dependent variables (CMJ, IMTP and CMJRJ). Descriptive data were presented for group-based comparisons, aligning individual data points to box and whisker plots and the data distribution. Subsequent Bonferroni post-hoc analyses were performed to follow-up significant between-group effects. Cohen’s *d* effect sizes (*d*) were determined by hand with outcomes defined as trivial (0.00 to 0.19), small (0.20 to 0.59), moderate (0.60–1.19), large (1.20–1.99) and very large (≥2.00) in accordance with established thresholds for determination [27]. Data were bootstrapped to 10000 samples, due to the improved confidence interval precision [28,29]. All statistical analyses were performed using JASP (JASP Team (2023). JASP (Version 0.18.1) [Computer software].). Alpha error probability was set at 0.05.

## Results

### Reliability

Descriptive data (mean ± SD) and within-session reliability statistics for each league for the CMJ, IMTP and CMJRJ data are presented in Tables 1–3, respectively. Data can be found in the supplementary material (Sup. 1–3 in S1 File).

**Table 1:** Descriptive data and within session reliability for the countermovement jump between leagues.

		CMJ height (m)	CMJ momentum (kg · m.s^-1^)	Countermovement depth (m)	Relative force at minimum displacement (N · kg^-1^)	Relative average braking force (N · kg^-1^)	Relative average propulsion force (N · kg^-1^)	Time to take-off (s)	mRSI (AU)
**Non-League**	**Mean ± SD**	0.37 ± 0.04	222.75 ± 26.10	-0.23 ± 0.04	28.59 ± 4.72	20.62 ± 3.51	23.64 ± 2.48	0.62 ± 0.10	0.62 ± 0.12
	**CV% (95% CI)**	3.40 (2.42-4.39)	1.68 (1.19-2.17)	7.41 (5.27-9.55)	6.32 (4.49-8.14)	7.99 (5.68-10.30)	3.10 (2.21-4.00)	8.94 (6.36-11.53)	9.21 (6.55-11.87)
	**ICC (95% CI)**	0.880 (0.815-0.938)	0.978 (0.961-0.988)	0.787 (0.660-0.878)	0.804 (0.689-0.888)	0.743 (0.602-0.850)	0.881 (0.804-0.934)	0.591 (0.407-0.749)	0.699 (0.542-0.822)
**National League**	**Mean ± SD**	0.39 ± 0.05	224.86 ± 28.27	-0.31 ± 0.06	25.76 ± 3.44	19.50 ± 2.36	21.38 ± 1.91	0.71 ± 0.11	0.56 ± 0.10
	**CV% (95% CI)**	2.94 (2.23-3.65)	1.45 (1.10-1.80)	5.54 (4.20-6.88)	4.20 (3.19-5.22)	4.61 (3.50-5.72)	2.27 (1.72-2.82)	5.76 (4.37-7.15)	6.36 (4.82-7.89)
	**ICC (95% CI)**	0.943 (0.910-0.966)	0.983 (0.973-0.990)	0.920 (0.874-0.952)	0.867 (0.796-0.919)	0.834 (0.748-0.898)	0.903 (0.849-0.942)	0.783 (0.677-0.865)	0.791 (0.688-0.870)
**League 2**	**Mean ± SD**	0.39 ± 0.06	228.40 ± 27.02	-0.29 ± 0.07	27.99 ± 4.65	21.03 ± 2.73	22.83 ± 2.79	0.68 ± 0.12	0.59 ± 0.13
	**CV% (95% CI)**	3.36 (2.35-4.38)	1.68 (1.17-2.19)	6.87 (4.79-8.95)	4.51 (3.15-5.87)	4.78 (3.34-6.23)	2.60 (1.82-3.39)	6.16 (4.30-8.02)	6.14 (4.28-8.00)
	**ICC (95% CI)**	0.962 (0.931-0.981)	0.983 (0.969-0.992)	0.948 (0.906-0.974)	0.906 (0.834-0.952)	0.834 (0.718-0.914)	0.950 (0.910-0.975)	0.837 (0.722-0.915)	0.911 (0.842-0.955)

SD = standard deviation, CV% = coefficient of variation percentage, CI = confidence intervals, CMJ = countermovement jump, mRSI = modified reactive strength index, AU = arbitrary units.

**Table 2:** Descriptive data and within session reliability for the isometric mid-thigh pull between leagues.

		Net peak force (N)	Relative net peak force (N · kg^-1^)	Net force at 100 ms (N)	Net force at 250 ms (N)	Relative net force at 100 ms (N · kg^-1^)	Relative net force at 250 ms (N · kg^-1^)
**Non-League**	**Mean ± SD**	1874.38 ± 360.03	22.87 ± 5.91	797.32 ± 312.41	1376.28 ± 302.79	9.41 ± 4.15	16.90 ± 4.88
	**CV% (95% CI)**	6.36 (4.52-8.19)	6.26 (4.45-8.07)	9.21 (5.97-12.45)	6.14 (4.37-7.92)	9.16 (5.94-12.39)	6.19 (4.40-7.97)
	**ICC (95% CI)**	0.864 (0.773-0.926)	0.926 (0.872-0.960)	0.766 (0.627-0.869)	0.838 (0.733-0.911)	0.806 (0.685-0.892)	0.923 (0.868-0.959)
**National League**	**Mean ± SD**	1959.32 ± 414.46	24.37 ± 5.65	654.58 ± 235.88	1363.82 ± 320.21	8.09 ± 3.14	17.06 ± 4.17
	**CV% (95% CI)**	5.26 (3.99-6.53)	5.29 (4.01-6.57)	11.37 (6.70-16.04)	7.35 (6.39-9.30)	11.59 (7.00-16.18)	8.31 (6.36-10.26)
	**ICC (95% CI)**	0.909 (0.859-0.944)	0.922 (0.878-0.952)	0.660 (0.501-0.802)	0.817 (0.726-0.885)	0.731 (0.585-0.856)	0.829 (0.744-0.893)
**League 2**	**Mean ± SD**	1920.64 ± 450.63	23.64 ± 6.57	664.29 ± 290.00	1211.82 ± 304.23	7.49 ± 3.46	14.95 ± 4.01
	**CV% (95% CI)**	9.73 (6.79-12.67)	9.63 (6.72-12.54)	10.03 (6.46-16.12)	8.74 (4.79-12.68)	10.67 (4.41-16.44)	6.91 (4.46-9.37)
	**ICC (95% CI)**	0.703 (0.527-0.838)	0.786 (0.644-0.886)	0.707 (0.507-0.910)	0.728 (0.528-0.790)	0.720 (0.598-0.918)	0.784 (0.601-0.926)

SD = standard deviation, CV% = coefficient of variation percentage, CI = confidence intervals.

**Table 3:** Descriptive data and within session reliability for the countermovement rebound jump between leagues.

		CMJ height (cm)	Rebound jump height (cm)	Rebound ground contact time (ms)	Rebound relative force at minimum displacement (N · kg^-1^)	Rebound RSI (AU)	Rebound relative average braking force (N · kg^-1^)	Rebound relative average propulsion force (N · kg^-1^)
**Non-League**	**Mean ± SD**	0.34 ± 0.03	0.37 ± 0.04	277.61 ± 77.49	45.60 ± 13.55	2.10 ± 0.61	32.43 ± 7.88	29.46 ± 5.87
	**CV% (95% CI)**	4.66 (3.31-6.00)	4.90 (3.48-6.32)	8.81 (5.40-12.22)	9.76 (6.08-13.45)	8.32 (4.76-11.88)	8.27 (5.30-11.23)	7.28 (5.17-9.38)
	**ICC (95% CI)**	0.710 (0.550-0.834)	0.796 (0.670-0.886)	0.795 (0.669-0.886)	0.733 (0.582-0.849)	0.800 (0.676-0.889)	0.810 (0.691-0.895)	0.811 (0.692-0.895)
**National League**	**Mean ± SD**	0.36 ± 0.05	0.34 ± 0.07	199.87 ± 28.42	58.20 ± 11.18	2.64 ± 0.43	39.89 ± 5.17	33.75 ± 3.95
	**CV% (95% CI)**	3.76 (2.85-4.66)	6.71 (4.85-8.58)	5.71 (4.33-7.09)	8.09 (6.14-10.05)	6.23 (4.73-7.74)	5.37 (4.08-6.67)	4.71 (3.57-5.84)
	**ICC (95% CI)**	0.865 (0.792-0.918)	0.780 (0.673-0.863)	0.764 (0.651-0.852)	0.752 (0.635-0.844)	0.806 (0.709-0.880)	0.751 (0.633-0.843)	0.847 (0.628-0.840)
**League 2**	**Mean ± SD**	0.36 ± 0.06	0.35 ± 0.08	195.25 ± 22.79	57.89 ± 9.06	2.74 ± 0.46	41.28 ± 5.32	34.31 ± 4.24
	**CV% (95% CI)**	4.00 (2.79-5.22)	6.00 (3.88-8.11)	6.42 (4.48-8.36)	7.93 (4.51-10.15)	6.67 (4.65-8.68)	6.55(4.57-8.54)	4.21 (2.94-5.49)
	**ICC (95% CI)**	0.837 (0.721-0.915)	0.838 (0.724-0.916)	0.716 (0.513-0.883)	0.817 (0.595-0.916)	0.789 (0.649-0.888)	0.784 (0.601-0.926)	0.848 (0.739-0.921)

SD = standard deviation, CV% = coefficient of variation percentage, CI = confidence intervals, CMJ = countermovement jump, RSI = reactive strength index, AU = arbitrary units.

The CMJ displayed poor to excellent relative reliability, and this was observed for all measures across all competitive leagues. Time to take-off had the lowest relative reliability within non-league soccer players. Moderate to excellent absolute reliability was observed for all CMJ measures across all competitive leagues (Table 1). The IMTP displayed moderate to good relative and absolute reliability for peak and relative net force and rapid force at 250 ms across competitive leagues. Moderate-poor absolute and moderate relative reliability was observed absolute and relative rapid force assessed at 100 ms (Table 2). Moderate to good relative reliability was observed for all CMJRJ measures for all competitive leagues, with moderate to excellent absolute reliability also observed (Table 3).

### Countermovement jump

**Fig 1:**
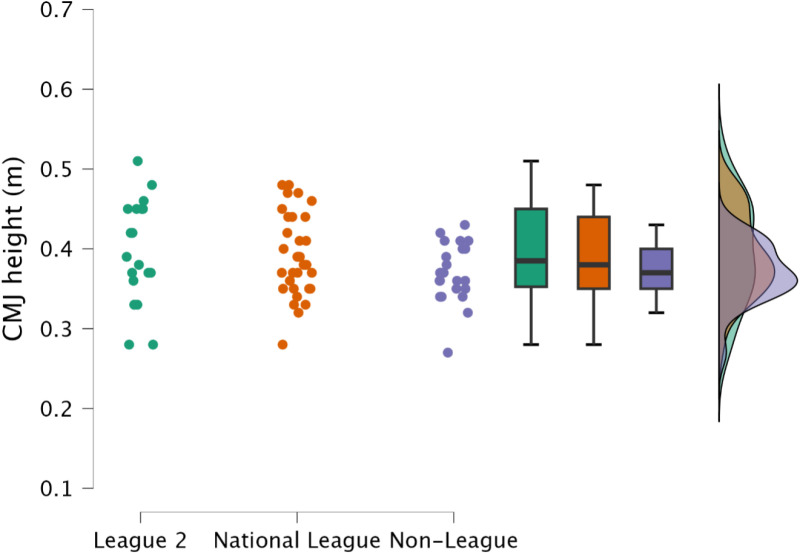
Comparisons of countermovement jump height between leagues, including individual data points, box and whisker plots and distribution of data.

Non-significant (*p* > 0.726), trivial to small (*d* = 0.13 to 0.32) differences were observed in the body mass between leagues. Similarly non-significant (*p* > 0.382), trivial to small (*d* = 0.05 to 0.47) differences were observed in CMJ jump height (Fig 1).

Significant (*p* ≤ 0.009), moderate to large (*d* = 0.94 to 1.25) differences were observed in the countermovement depth, with non-league players having performed a shallower countermovement than EFL2 and NL soccer players, a non-significant small difference was observed between EFL2 and NL for countermovement depth (*p *= 0.832, *d* = 0.31). Non-significant, trivial to moderate differences were observed in the CMJ jump momentum (*p *= 1.000, *d* = 0.05 to 0.13), relative average braking force (*p *≥ 0.514, *d* = 0.01 to 0.39), relative average propulsion force (*p *≥ 0.229, *d* = 0.39 to 0.55), CMJ time to take-off (*p *≥ 0.265, *d* = 0.31 to 0.53) and mRSI between leagues (*p *≥ 0.383, *d* = 0.10 to 0.42).

### Isometric mid-thigh pull

**Fig 2:**
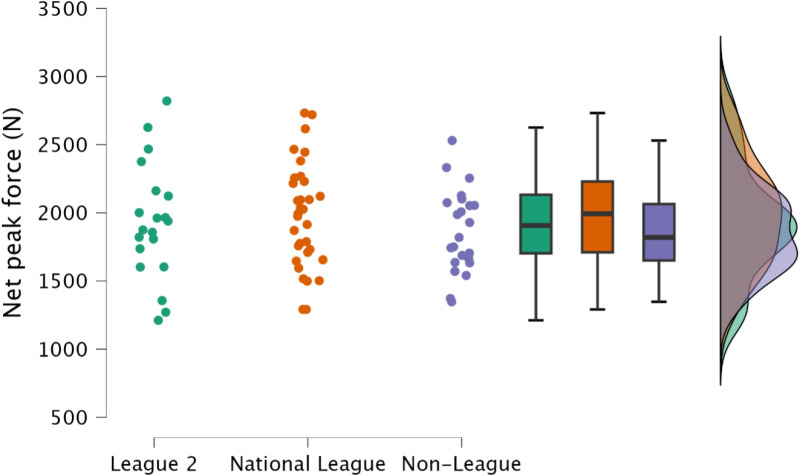
Comparison of absolute isometric mid-thigh pull peak net force between leagues, including individual data points, box and whisker plots and distribution of data.

**Fig 3:**
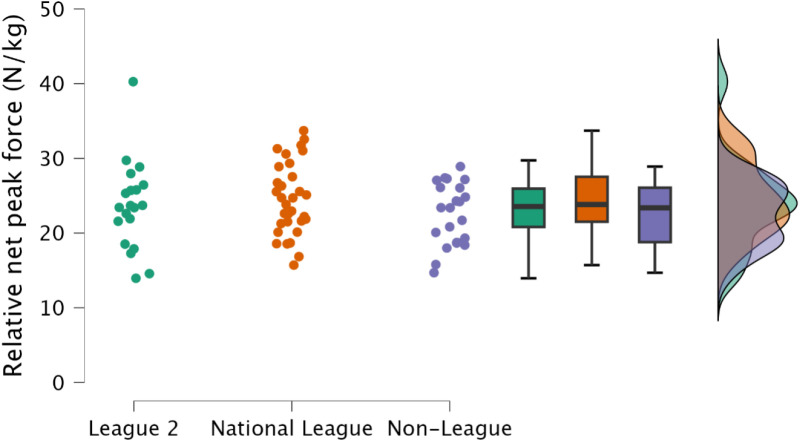
Comparison of relative isometric mid-thigh pull peak force between leagues, including individual data points, box and whisker plots and distribution of data.

Non-significant, trivial to small differences were observed in both absolute (*p *≥ 0.810, *d* = 0.13 to 0.30), and relative peak net force (*p *≥ 0.396, *d* = 0.16 to 0.41) (Figs 2 & 3, respectively).

**Fig 4:**
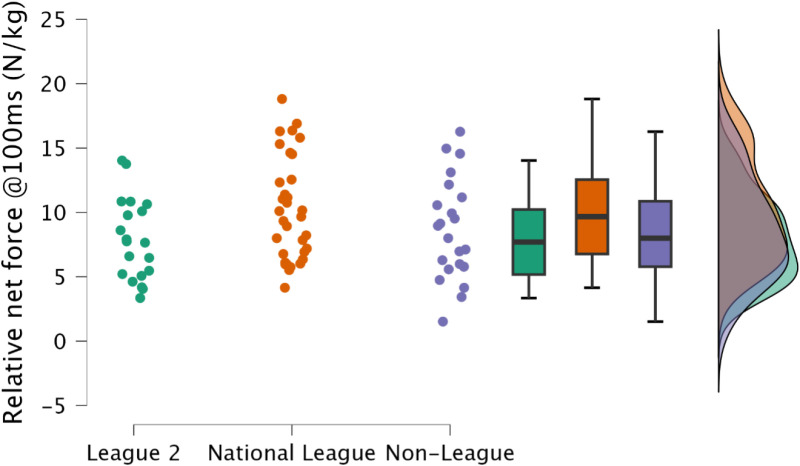
Comparison of relative isometric mid-thigh pull peak force at 100 ms between leagues, including individual data points, box and whisker plots and distribution of data.

**Fig 5:**
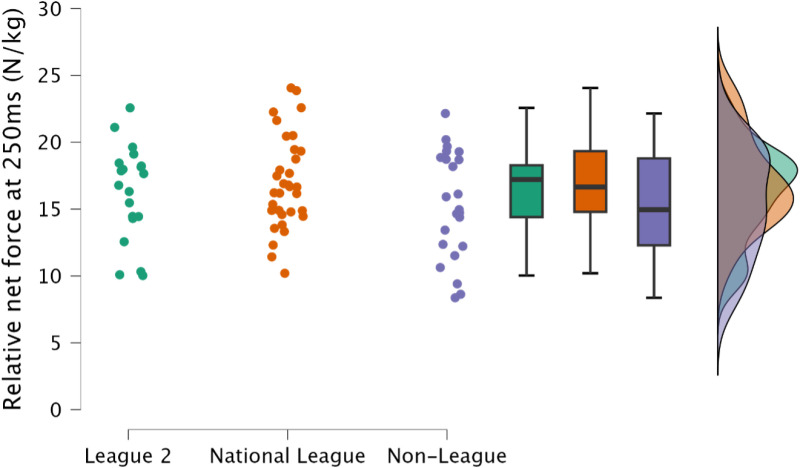
Comparison of relative isometric mid-thigh pull peak force at 250 ms between leagues, including individual data points, box and whisker plots and distribution of data.

Non-significant, trivial to moderate differences were observed in relative peak force at 100 ms (*p *≥ 0.092, *d* = 0.18 to 0.63), and 250 ms between leagues (*p *≥ 0.307, *d* = 0.19 to 0.45) (Figs 4 & 5, respectively).

### Countermovement rebound jump

**Fig 6:**
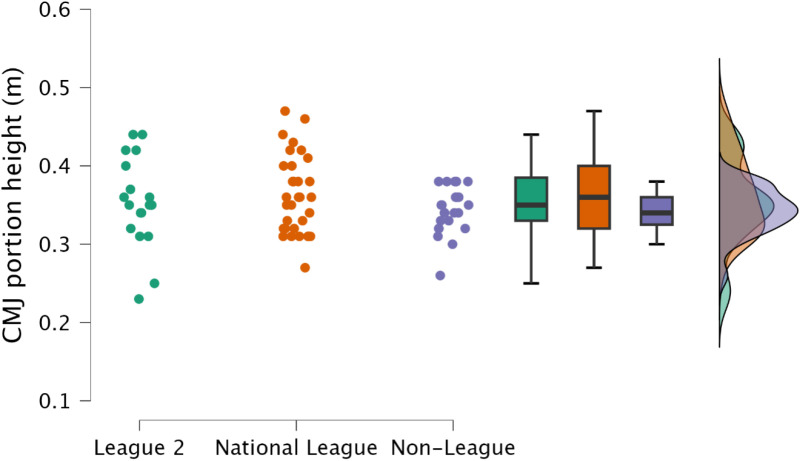
Comparison of countermovement jump height within the countermovement rebound jump between leagues, with individual data points, box and whisker plots and distribution of data.

Non-significant, trivial to small differences were observed in the CMJ-portion jump height (*p *≥ 0.440, *d* = 0.17 to 0.39) (Fig 6).

**Fig 7:**
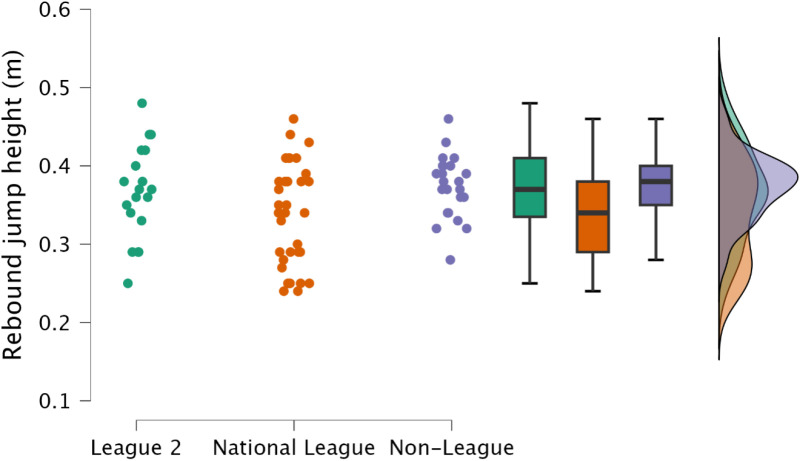
Comparison of rebound jump height within the countermovement rebound jump between leagues, including individual data points, box and whisker plots and distribution of data.

Non-significant, trivial to moderate differences in rebound jump height (*p *≥ 0.077, *d* = 0.13 to 0.48), and rebound jump momentum (*p *≥ 0.465, *d* = 0.30 to 0.41) were observed between EFL2 and non-league (Fig 7). A non-significant, yet moderate (*p *= 0.077, *d* = 0.62) difference was observed between NL and non-league for rebound jump height, while rebound jump momentum had a significant (*p* = 0.032) and moderate (*d* = 0.71) difference between NL and non-league. National league footballers had the lowest rebound jump height of all three groups (Table 3, Fig 7).

**Fig 8:**
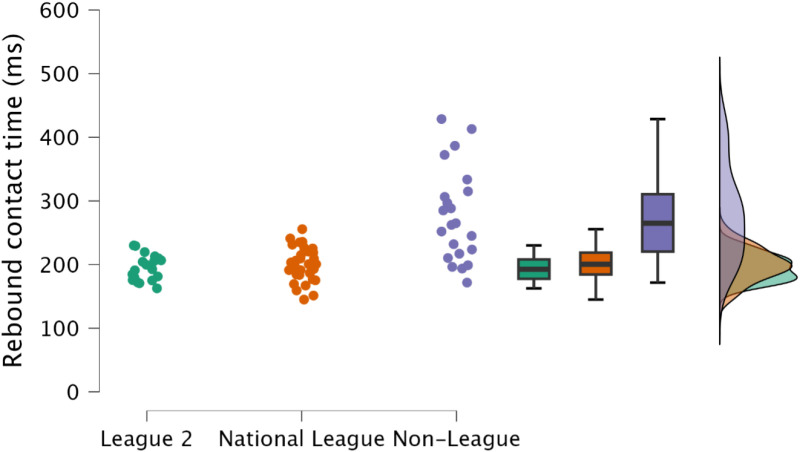
Comparison of rebound contact time within the countermovement rebound jump between leagues, with individual data points, box and whisker plots and distribution of data.

**Fig 9:**
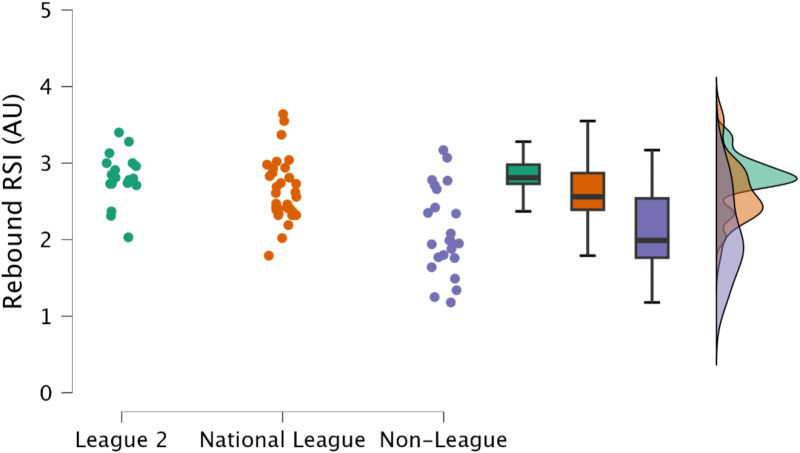
Comparison of rebound reactive strength index time within the countermovement rebound jump between leagues, including individual data points, box and whisker plots and distribution of data.

Non-significant (*p *≥ 0.536), trivial and small differences (*d* = 0.11 to 0.39) in rebound contact time and rebound RSI, respectively, were observed between EFL2 and NL. However, non-league soccer players had a significantly (*p* ≤ 0.001, *d* = 1.20 to 1.85) longer rebound contact time (Fig 8) and lower rebound RSI (Fig 9) to a large magnitude when compared NL and EFL2 soccer players.

**Fig 10:**
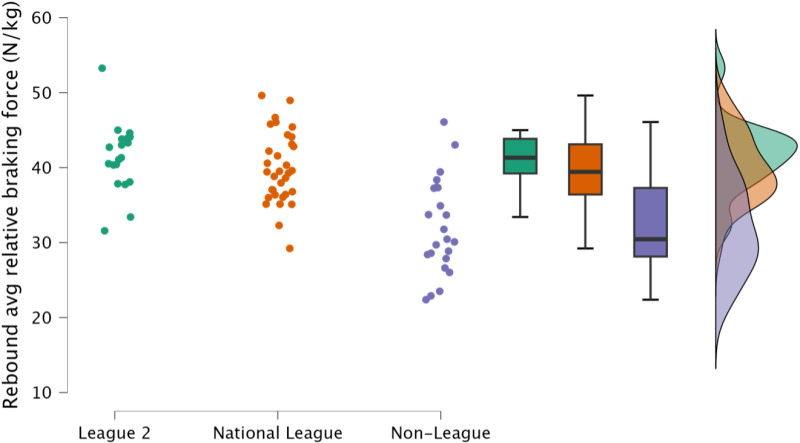
Comparison of rebound average relative braking force within the countermovement rebound jump between leagues, including individual data points, box and whisker plots and distribution of data. Avg = average.

**Fig 11:**
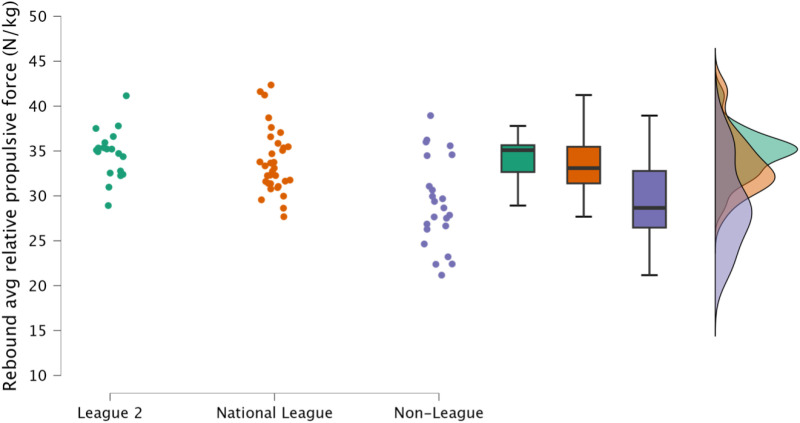
Comparison of rebound average relative propulsion force within the countermovement rebound jump between leagues, including individual data points, box and whisker plots and distribution of data. Avg = average.

Non-significant, small differences in rebound average relative braking and propulsion forces were observed between EFL2 and NL (*p* = 1.000, *d *= 0.23 to 0.28). However, in contrast, non-league soccer players achieved significantly *(p *≤ 0.001) lower forces in this task, with a moderate to large effect noted in contrasts made to both EFL2 and NL (*d* = 1.18 to 1.76, Figs 10 & 11, respectively).

## Discussion

The results of the present study highlight that there are no meaningful differences between ballistic and maximal strength qualities between levels of competition in the English leagues. However, meaningful differences in the reactive qualities were observed between tiers, with non-league soccer players being less able to adopt the required stiff strategy to performance, resulting in a meaningfully longer ground contact time and reduced relative braking and propulsion forces during the rebound portion of CMJRJ. A robust battery of physical performance tests to create a global performance profile for athletes has been suggested to include measures of ballistic, reactive and maximal strength capabilities [30–32], this could be achieved by using force plates and performance tests such as the CMJ, CMJRJ and IMTP. Determining physical performance characteristics of soccer players between levels of competition can support coaches to determining training schedules to enable targeted development of required physical characteristics and thereby enhance physical performance in competitive situations. It can also aid coaches working within the lower tiers of English football with recruitment and talent identification, with player selection potentially dependent on reactive ability due to associations with scoring situations [1,3,33].

Reactive qualities, as determined by the fast stretch shortening cycle and an athlete’s ability to effectively their elastic tissues (such as the Achilles tendon) to perform purposeful work [34,35], are a key determinant for rapid, explosive actions, such as sprinting and changing direction [11,36,37]. Within the present study, rebound jump ability was a discriminating factor, identifying variability in attributes between players performing at different levels of the tier system; specifically, the distinction was an ability to perform the rebound jump in a short time frame (<250 ms) (Figs 8, 9). While the outcome measure (rebound jump height) was similar between leagues with only trivial to small differences observed (Fig 7), there were clear differences in the ground contact time. Therefore, non-league players were adopting a compliant strategy to emphasise the rebound jump height, yet at the cost of sacrificing speed. This observation is also supported by the moderate to large decreases in rebound average relative braking and propulsion forces (Figs 10, 11). Despite consistent and standardised cueing, whereby participants were instructed to “*jump as fast as possible*” [22,38], non-league players were unable to achieve a ground contact time of <250 ms. There is currently limited research on using the CMJRJ as a measure of fast stretch shortening cycle ability. Xu and colleagues [34,38] recently examined the between-session reliability and the joint work and joint contribution of the CMRJ. Consistent with the results of the present study, the CMJRJ was deemed a reliable method of assessing fast stretch shortening cycle ability [ICC = 0.94 (95% CI = 0.89 to 0.97), CV% = 8.13 (95% CI = 6.07 to 10.19] [38]. However, similar to the non-league players within the present study, the sport science student population sampled in the work of Xu et al. [38] were also unable to achieve a ground contact time of <250 ms (Table 3), although this could have also been due to how the tasks were coached. This highlights the performance of a fast stretch shortening cycle task, where the athletes are required to adopt a stiff ankle dominant strategy to perform sprints and shallow directional changes (e.g., side-step cuts and crossover cuts) [39], which could be a determining factor in successful soccer performance (for instance required for goal scoring situations) [1–3].

Maximal lower body strength is a crucial physical quality required for team sports athletes [14], with gains in maximal lower body strength facilitating improvements in athletic performance tasks such as sprinting, jumping, change of direction [14], and sport specific tasks [14,40,41]. Similarly, increases in lower body strength are associated with injury risk mitigation [42,43], with the greatest relative IMTP scores resulting in the lowest annual injury rate [43]. Specifically, with regards to soccer, increased lower body strength results in decreased markers of muscle damage following soccer match play [44]. Hence, both factors would result with the athlete having increased availability for competition and training, potentially enabling greater global athlete development (sport technical qualities, sport-specific physical qualities and general physical qualities) [14]. Despite no meaningful difference between leagues for IMTP net peak force (Figs 2 and 3), demonstrating that maximal strength may not be the underlying physical qualities required to increased competitive soccer success, it is noteworthy that most soccer players observed within the present study across all leagues would be considered to have substandard levels of strength, with average peak relative net IMTP forces of <30 N/kg (Table 2). Within Spanish footballers, Soriano et al. [8] reported small to moderate differences between national and regional players for absolute and relative peak force (IMTP), although in contrast to the present study, the authors reported gross force. Interestingly, when represented as net forces, these would be consistent with the present study, with average relative peak force <30 N/kg. Clearly this indicates that soccer players should dedicate time to enhancing lower body strength. Investment of time in this area could extend playing career, supplementing both training and competition. The IMTP can also be used to assess rapid isometric force [45,46], an essential physical quality in a sport where the acceleration of one’s mass is integral to successful performance [47,48]. Consistent with relative net peak force, there was no observed difference between leagues in rapid isometric force at either 100 ms or 250 ms (Figs 4 and 5). This is likely explained by there being no difference in relative net peak force, as observed between strength-matched males and females, where relative peak IMTP force explained rapid force production capabilities in the IMTP [46]. As previously identified, the soccer players included within the present study would be classified as substandard in terms of relative strength, therefore increases in maximal strength should be the primary training goal.

Ballistic capabilities appeared not to be differentiated between the leagues, with only a trivial to small difference in CMJ outcome (jump height) (Fig 1). Interestingly however, despite the lack of differences in CMJ outcome, non-league soccer players achieved this jump height by performing a shallower countermovement (Table 1). This could be a positive performance, whereby the non-league soccer players would be performing a greater amount of work over a shorter range of motion, however, as there was no difference in CMJ time to take off and average braking and propulsion forces, it could be suggested that this is not a desired performance as the athletes are taking a similar duration to perform a shallower countermovement [49]. This further supports the requirement for strength development, especially within the non-league players but across all leagues, as increased lower body strength following a strength training block improved jump performance via increases in jump outcome through changes in force, velocity and power, without changing displacement [50,51]. Moreover, weaker individuals would experience greater changes in slow stretch shortening cycle ability through strength training and not power training [50,52,53]. Following a period of strength training, non-league soccer players could increase their CMJ outcome without altering their displacement [50]. Whilst positive outcomes in match-play are dependent on a number of events it is plausible that such alterations in physical function could facilitate a greater incidence of desirable outcomes during competitive situations (i.e., goal scoring situations) [1–3]. Moreover, improving jump performance by changing unweighting, braking and propulsive velocity with only minimal changes in displacement would result in decreased time to take-off, therefore, if players improved jump outcome (i.e., height) and decreased time to take-off, they would be able to jump higher and leave the ground quicker than their counterparts in the higher leagues which could offer a competitive advantage, for example, in goal scoring situations.

Within English and European soccer, there is a lack of focus on strength development with teams prioritising high-velocity power-based exercises in-season typically adopting an emphasis-based resistance program within a reduced focus on strength training [15,54], which is likely due to the negative connotations within European soccer with strength training resulting in delayed onset muscle soreness [55]. This is despite evidence to utilise the various low volume, micro-dosed approaches to resistance training to enhance strength without causing delayed onset muscle soreness and inducing fatigue [56,57]. This is further supported by majority of soccer strength and conditioning practices within the United Kingdom not aligning with scientific guidelines, with body weight training being believed to have a similar ability to develop strength and power as free weight training [15]. Within the present study, observations were made of proportionately low-level teams within the English soccer league system, including fourth, fifth and seventh tier teams, it could be suggested that the lack of focus on strength-based training could be due to a fundamental lack of resources, including not having dedicated strength and conditioning coaches, with professionals with a remit for other specialist roles (i.e., sport science, physiotherapy and/or sport medicine) often required to deliver training sessions and/or design interventions. Hence, staff maybe working outside of their specialty and not be adept in developing targeted strength and conditioning programmes to emphasise maximal lower body strength within the constraints of an English soccer playing frequency. A secondary factor could be a lack of physical resources to develop strength, i.e., a lack of time and facilities, which could be leading to coaches prioritising speed and power development over maximal strength development, and self-directed programming (where athlete perform on their own) typically has lower uptake and compliance than a coached programme [58–60].

The present study is not without its limitations, firstly all testing took place within the first week of pre-season highlighting that the players may not be at their peak physical condition ready for competition after a period of off-season training. This approach was merited, as it ensured a standardised rested state was achieved across all leagues but may not be truly representative of the required fitness for each league. If testing took place during the first week of the competitive ‘in-season’, it would be expected that athletes would be close to their peak physical fitness and could be hypothesised that further differences in ballistic and strength capabilities might be observed. However, professional clubs are protective of their players, and it is not always feasible nor practical to intervene close to the start of a season. In any case, further investigation is needed. Furthermore, there is currently limited information on the match demands of the leagues involved within the present study, if there is limited difference between the leagues then this could explain the lack of difference in physical characteristics. Within Norwegian soccer, between tiers 1, 2 and 4 there is a significant and moderate increase in sprint running and the number of accelerations [7], although when this was observed by positions only attackers and central defenders showed this trend. However, the authors only considered null hypothesis testing within their results, despite small-large effect sizes observed for all positions [7]. It should also be noted, that while the results of the present study provide a robust physical performance profile on force generating capabilities, physiological capacity (i.e., aerobic capacity) is also a key determinant within soccer [61,62], hence further investigation is required to determine if there are any differences in aerobic performance between leagues either using lab or field based performance assessments.

## Conclusions

At different levels of English soccer players, differences are not apparent within ballistic or maximal isometric assessments. However, there were meaningful differences in the reactive qualities, specifically the kinematic and kinetics of the rebound jump portion of CMJRJ, with meaningfully greater ground contact time and decreased braking and propulsion forces. Interestingly, regardless of league, most of the soccer players who participated within the present study would be categorised as weak (relative net peak force <30 N/kg). If appropriate strength and conditioning practices were adopted to increase strength, via the inclusion of resistance training guidelines which could be a micro-dosing approach, improvements would be expected across other measures of physical and potentially technical performance via increased playing and training time with decreased risk of injury.

## Supporting information

S1 File League CMJ Differences. (ZIP)
